# An overview to nanocellulose clinical application: Biocompatibility and opportunities in disease treatment

**DOI:** 10.1016/j.reth.2023.10.006

**Published:** 2023-11-16

**Authors:** Kosar Malekpour, Ali Hazrati, Arezou Khosrojerdi, Leila Roshangar, Majid Ahmadi

**Affiliations:** a Department of Immunology, School of Medicine, Iran University of Medical Sciences, Tehran, Iran; b Department of Immunology, School of Medicine, Tehran University of Medical Sciences, Tehran, Iran; c Infectious Disease Research Center, Birjand University of Medical Sciences, Birjand, Iran; d Stem Cell Research Center, Tabriz University of Medical Sciences, Tabriz, Iran

**Keywords:** Bionanomaterials, Nanocellulose, Nano-lignin, Tissue engineering, Tissue repair, Drug delivery

## Abstract

Recently, the demand for organ transplantation has promptly increased due to the enhanced incidence of body organ failure, the increasing efficiency of transplantation, and the improvement in post-transplant outcomes. However, due to a lack of suitable organs for transplantation to fulfill current demand, significant organ shortage problems have emerged. Developing efficient technologies in combination with tissue engineering (TE) has opened new ways of producing engineered tissue substitutes. The use of natural nanoparticles (NPs) such as nanocellulose (NC) and nano-lignin should be used as suitable candidates in TE due to their desirable properties. Many studies have used these components to form scaffolds and three-dimensional (3D) cultures of cells derived from different tissues for tissue repair.

Interestingly, these natural NPs can afford scaffolds a degree of control over their characteristics, such as modifying their mechanical strength and distributing bioactive compounds in a controlled manner. These bionanomaterials are produced from various sources and are highly compatible with human-derived cells as they are derived from natural components. In this review, we discuss some new studies in this field. This review summarizes the scaffolds based on NC, counting nanocrystalline cellulose and nanofibrillated cellulose. Also, the efficient approaches that can extract cellulose with high purity and increased safety are discussed. We concentrate on the most recent research on the use of NC-based scaffolds for the restoration, enhancement, or replacement of injured organs and tissues, such as cartilage, skin, arteries, brain, and bone. Finally, we suggest the experiments and promises of NC-based TE scaffolds.

## 1 Introduction

Tissue engineering (TE) consists of integrating and using several biological and medical disciplines that aim to replace damaged tissues and repair and improve them [1]. This method's obstacles are the lack of suitable biological materials and defects in intercellular interactions [2,3]. These insufficient growth factors stimulate these interactions and also the inability to control cellular functions, including biological, mechanical, electrochemical, and their various properties [4]. The use of NPs has overcome many of these limitations in TE, making it an attractive field for repairing and regenerating various tissues, including skin, bone, and cartilage [5]. The advantages of NPs include their small size, high surface-to-volume ratio, easy integration with biological membranes, and the ability to access particles of any size [6]. NPs perform different functions depending on the type and their characteristics, which generally leads to creating an efficient nanomedicine field [7]. Besides, unstable bioactivity, poor solubility, and short circulation half-life of biomolecules (cytokines, growth factors, inhibitors, medicines, genes, etc.) and contrast agents have established NPs as one of the most promising options for bioactive compound distribution and assessment for applications [1,8]. These NPs have a wide range of biological applications, such as active pharmaceutical drug delivery [9,10].

TE's three essential elements are seeding cells, regulatory factors, and scaffolds [11]. Scaffolds are three-dimensional structures that are used to culture cells three-dimensionally to provide and mimic various cellular connections and growth factors based on what is happening in body tissues [12]. Using different growth factors and inserting them into scaffolds can change the behavior of cells as desired and guide their differentiation and growth in a controlled manner [13,14]. Interestingly, nanotechnology has modified traditional and simple TE approaches, providing more powerful and advanced systems. Other nanoscale products, such as nanofibers (NFs) and nanopatterned surfaces, have been employed to control cellular functions in the TE field with NPs. Using combined therapeutic and imaging frameworks, incorporating novel biocomposites with excellent spatiotemporal control within scaffolds, the modifying release of several therapeutic compounds, particularly growth factors, to direct stem cell fate and morphogenesis, adjusting the mechanical strength of scaffolds for hard tissue applications, and minimizing toxicity and increasing biocompatibility through tissue-specific delivery are just a few of the many uses of NPs in TE [15]. As mentioned, nanocellulose has a high biocompatibility potential. The biocompatibility of a substance in the body is related to the reactions after its transplantation to the body that do not cause harmful responses [16]. Also, due to the fact that there is no cellulose-degrading related enzyme in the human body, this type of material is not decomposed in the body and can continue to function for a long time [17]. Hemocompatibility, also named blood compatibility, is another issue that should be considered in the biocompatibility of a material that is in contact with blood, including synthetic vessels [18]. Various histological and immunological investigations showed that in mice receiving nanocellulose and bacterial cellulose transplants, there is no difference in the proportion of the immune cell population in the bone marrow compared to the control group [19]. Also, the examination of hemocompatibility in mice receiving bacterial cellulose through plasma recalcification time (PRT) and whole blood clotting tests shows the compatibility of this material for therapeutic applications. Various methods have been used to increase the biocompatibility of cellulose [20]. One of them is the use of this material in nano size. It is also possible to increase their hemocompatibility, biocompatibility, and biological activity by oxidizing nanocellulose fibers using TEMPO [21]. Also, various nanocellulose biosynthesis methods can affect their biocompatibility [17]. Therefore, the use of methods that lead to maintaining the biocompatibility of nanocellulose and also increase their biological activity can increase their therapeutic efficiency.

Thus, these nanostructures have been used to serve various functions in TE, ranging from enhancing electrical, biological, and mechanical properties to gene delivery, DNA transfection, viral transduction, and patterning of cells to facilitate the growth of various types of tissues to molecular detection and biosensing [3,22]. Table 1 summarizes the application of some biomaterials in regenerative medicine and TE.

**Table 1:** Biomaterials Use in Regenerative Medicine.

Biomaterials	Targeted Tissues	Model of Study: In Vitro/In Vivo	Structure	Mode of Application	Ref
Chitosan	Blood vessel, Bone, Cartilage, Intervertebral disc, Skin	More in the form of In vitro and in some cases in vivo	Copolymer of (1–4)-2-acetamido-D-glucose and -(1–4)-2-amino-Dglucose units	Nanofiber, scaffold, Nano hybrid, injectable gels, hydrogels, microcarrier	[[23], [24], [25], [26], [27]]
Collagen	Bone, Cartilage, Muscle, Cancer,	In vitro	Mainly composed of glycine, proline, or hydroxyproline and form triple helix	Scaffold, hydrogels, matrices	[[28], [29], [30], [31]]
Silk Fibroin	Bone, Cartilage, Tympanic membrane	More in the form of In vitro	Semi crystalline structure composed of fibroin (75 %) and sericin (25 %)	Micro carrier, scaffolds	[[30], [31], [32]]
Alginate	Germ cells, Bone, Pancreas, Cancer	In vitro and In vivo	Copolymer of α-L-guluronic acid and β-D-mannuronic acid	Crosslinked, gel encapsulate, microbeads,	[[33], [34], [35]]
Poly lactic acid (PLA)	Bone	In vitro	Thermoplastic polyester with backbone formula ₙ or [–CHCO–] ₙ	Scaffolds	[36,37]
Poly lactic-co glycolic Acid (PLGA)	Bone, Cartilage, Nerve	In vitro and In vivo	Copolymer of poly lactic acid and poly glycolic acid	Nanoparticles, scaffold, microcarriers	[[38], [39], [40]]
Agarose	Cartilage, Nerve, Skin	In vitro and In vivo for Cartilage and skin, In vitro Nerve	Composed of the unit of D-galactose and 3.6-anhydro-L-galactopyranose	Nanostructured, Scaffold, hydrogels	[[41], [42], [43]]

Since NC, β-1, 4-glucose in the molecular chain has high mechanical strength [44], tailorable surface modification [45], good hydrophilicity [46], and excellent biocompatibility [47]. Due to these characteristics, NC is receiving a lot of interest, particularly in the biomedical industry. It can be mixed with polymers to create composites, which could be used to make TE scaffolds, drug carriers, wound dressings, and other things [[48], [49], [50]]. Here, we are committed to integrating the applications of NC in TE technologies, including the repairing and replacement of bone, skin, vascular, neural, and cartilage tissues. Also, the efficient methods to extract high-purity cellulose are discussed. Finally, we propose the trials and possibilities of NC-based TE scaffolds.

## 2 Nanocellulose and nano-lignin

Nanocellulose can be classified into three main types in terms of morphology, particle size, crystallinity, and some properties due to differences in sources and extraction methods. Nanocrystalline cellulose, nanofibrillated cellulose, and bacterial nanocellulose (Table 2) [51]. Nanocrystalline cellulose is usually extracted from cellulose fibrils by hydrolase acid, but nanofibrillated cellulose is extracted from cellulose fibrils by mechanical methods [52]. Compared to nanocrystalline cellulose, nanofibrillar cellulose has a longer length with an aspect ratio (length to diameter), a large surface area, and a large amplitude of hydroxyl groups that are easily accessible for surface modification [53]. However, bacterial nanocellulose is different from the other two groups and is produced by the production and accumulation of low molecular weight sugars by bacteria (mainly gluconactobacter xylinus) in a process that takes several days to two weeks [54,55]. The use of nanocellulose in polymer-based three-dimensional cell scaffolds has been developed to support important cellular activity in various cell cultures [56]. In fact, scaffolds can mimic extracellular matrix functions and play a role in intercellular communication and cellular biological processes [57,58]. NC-based scaffolds are suitable candidates for TE due to their characteristics, including high biocompatibility, ability to absorb and retain water, and chemical-mechanical properties. To produce NC-based scaffolds, four methods are usually used, including electrospinning, freeze drying, 3D printing, and solvent casting. The biocompatibility features of these scaffolds have led to their use in the repair of different tissues and organs [59]. Today, nanocellulose -based scaffolds are used to study human cancer tissues more accurately, cell cycle progression in these tissues and their cells, study gene expression, cellular connections, and their effect on tumor tissue growth [60]. Cellulose-based biocompatible materials can be used to produce efficient scaffolds for human cell culture due to their unique properties, such as non-cytotoxicity, porous structure, suitable three-dimensional shape, and desirable mechanical properties [61]. 3D cellulose scaffolds and their hybrids with chitosan, alginate, and agarose have been developed for various applications in TE [60]. Recently, the application of these scaffolds has been reported in pharmacies for drug delivery [62,63] and in regenerative medicine using transplantable scaffolds to treat cardiovascular and lumbar intervertebral disc diseases [64].

**Table 2:** Different types of nanocellulose and their characteristics.

Nanocellulose type	Typical sources	Formation	Average size	Crystalline Nature	Applications
Nano or microfibrillated cellulose (NFC/MFC)	Wood, sugar beet, potato tuber, hemp, flax	It can be extracted from cellulose chains using mechanical process to cleavage the fiber into nanometer size in diameter.	Diameter: 5–60 nm Length: several micrometers	Contains amorphous regions, therefore, less crystalline	Biomedical (tissue engg, scaffolds), Hydrogels, Paper and film industry, Electronic and Energy sector
Nanocrystalline cellulose (NCC)	Wood, cotton, hemp, flax, wheat straw, mulberry bark, ramie, Avicel, tunicin, cellulose from algae and bacteria	It can be extracted from cellulose chains using acid hydrolyzed amorphous region and left only crystalline region.	Diameter: 5–70 nm Length: 100–250 nm (from plant celluloses)	Higher crystalline nature than CNFs	Reinforcement, Pharmaceutical, Biological and agricultural sector
Bacterial nanocellulose (BNC)	Low-molecular weight sugars and alcohol	Bacterial synthesis	Diameter: 20–100 nm Different types of nano-fiber networks	Highly crystalline in nature	Biomedical implants, Food packaging, Aerogels, Cosmetics

Lignins are formed through phenolic oxidative coupling processes in the plant and are the major class of natural products present in the natural kingdom. Three monolignols—p-coumaryl, p-coniferyl, and sinapyl alcohols—produced from l-phenylalanine by the main phenylpropanoid pathway—were dehydrogenatively polymerized to generate these phenolic structures. In the cell walls of lignified plants, some polysaccharides join with lignin to produce lignin-carbohydrate complexes (LCCs) [65]. Lignin is a large molecule or reproducible biopolymer and has several functional groups, such as hydroxyl and aliphatic hydroxyl, thiol, and phenolic hydroxyl [66]. Lignin is the main biopolymer, along with cellulose, which separates more from wood. How to separate and identify pure lignin has not been fully described in any study. Nowadays, researchers have focused on synthesizing nano-lignin and its use in applied fields such as pharmacy, TE, industry, etc. [67]. Lignin protects the polysaccharide components by forming a matrix layer in the cell walls of plants. Lignins are composed of different types and are divided into three main groups: grass lignin, softwood lignin, and hardwood lignin. Although most lignins are separated from wood, they are usually considered enzymatically released as mild wood lignin (MWL) [68]. Usually, the composition of lignin varies from species to species and is represented by the chemical formula (C_31_H_34_O_11_)_n_ [69]. This compound has antioxidant and antimicrobial properties that have affected its clinical use. The functional groups of lignin structure provide an efficient interaction with secondary metabolites or reducing agents and facilitate the formation of a series of desired nanostructures. The physicochemical properties of these nanostructures must be confirmed by electron spectroscopy, X-ray photoelectron spectroscopy, infrared spectroscopy, contact angle measurement, and elemental analysis [70].

## 3 Therapeutic application of nanocellulose and nano-lignin

In the field of TE, the section on neural TE is one of the most complex and challenging types. The use of bacterial cellulose to adhere mesenchymal stromal/stem cells (MSCs) results in neurotrophic secretion increases. Also, it increases the therapeutic potential of MSCs in neuronal tissue regenerative medicine in laboratory studies [71,72]. The results of in vitro studies have also shown that bacterial cellulose is well compatible with Schwann cells and has no pathological or harmful effects. The point is that the above studies have all been studied on a short-term basis and on tun animals, and their impact on larger animals and even humans requires more and more gold studies [73]. Table 3, Table 4 summarize the clinical trials and new studies on the nanocellulose application in regenerative medicine and TE, respectively.

**Table 3:** Nanocellulose based clinical trials in skin-related disease treatment.

Study title	Status	Intervention model	Sponsor	Participants	NTC Number
New Treatment for Donor Sites	Withdrawn	Parallel Assignment	The University of Texas Medical Branch, Galveston	0	NCT00591916
NFC Dressing for Skin Graft Donor Sites	Completed	Single Group Assignment	UPM Biomedicals	33	NCT03980600

**Table 4:** A number of studies have used nanocellulose in tissue engineering.

Study name	Tissue	Type of nano cellulose	Model of Nanocellulose	Year	Ref.
Development and characterization of bacterial nanocellulose loaded with Boswellia serrata extract containing nanoemulsions as natural dressing for skin diseases	Skin	bacterial nanocellulose	nanoemulsion	2020	[74]
Structural and mechanical characterization of crosslinked and sterilized nanocellulose-based hydrogels for cartilage tissue engineering	Cartilage	Nano-fibrillated and Nanocrystalline cellulose	Crosslinked by Ccl2	2019	[75]
Cross-linked gelatin-nanocellulose scaffolds for bone tissue engineering	Bone	Nano-fibrillated cellulose	Crosslinked and scaffold forming	2020	[76]
Nanocellulose reinforced gellan-gum hydrogels as potential biological substitutes for annulus fibrosus tissue regeneration	Intervertebral disc	indeterminate	Hydrogel	2017	[77]
Composite aerogels based on dialdehyde nanocellulose and collagen for potential applications as wound dressing and tissue engineering scaffold	Skin	Nano-fibrillated cellulose	Crosslinked	2014	[78]

On the other hand, the data have confirmed that different types of nanocellulose s are not cytotoxic [79,80]. A fundamental application of biomaterials in the nanoscale is essential for designing a new scaffold that assembles the natural cellular environment. Nanoscale variation in the nanocellulose scaffold structures can have a macroscale effect on tissue reaction, for example, cellular adhesion, proliferation, differentiation, spreading, and migration. It is well known that the extracellular matrix (ECM) has a critical role in cell attachment and cellular biophysical, biochemical, and biomechanical interaction [81].

The wound-healing application of nanocellulose is reported in several pathophysiological events such as tissue regeneration, repair, angiogenesis, and reconstruction take place. For example, in burned patients and patients undergoing oncological treatment. Despite current advances in wound treatment, optimal approaches are lacking to promote wound healing (Table 5) [82]. Specific data indicate that nano cellulose-based materials as proper scaffolds can promote osteogenesis, cartilage repair, and vascular, neural, skin, and wound healing. The data demonstrated that the synthesis of cellulose-based biomaterial is a firm occurrence of good with high tissue integration regeneration in in-vivo studies. To overcome the limitations of intact nanocellulose, chemical modifications are critical and depend on the fabrication of the scaffold using cellulose derivatives MC, CMC, HPMC, HPC, and i.e., for the aim of tissue regeneration.

**Table 5:** Bacterial derived cellulose applied to wound healing.

Bacterial NC type	Reinforcement material	Synthetic strategy	Dose	Intervention time	Application type	Year	Ref.
BC/collagen	Collagen	In-situ	Scaffold diameters = 20 mm diameter × 4 mm height	24 h	Wound dressing and Tissue engineering	2011	[83]
OBC	Oxidized BC, chitosan, collagen	Ex-situ	58 mg	30 days	Hemostasis and wound healing	2020	[84]
Regenerated bacterial cellulose	Zinc oxide nanoparticles	Regeneration	15 mm diameter × 2 mm thickness	24 h	Antibacterial	2014	[85]
HACC/BC	Quaternized chitosan	Ex-situ	1, 3, and 5 mg/mL	3 days	Antibacterial, biocompatibility	2020	[86]
RBC-TiO2	Titanium dioxide nanoparticle	In-situ and regeneration	2 cm × 2 cm	24 h	Antibacterial	2015	N/A

### 3.1 Neural tissue

According to reports from various studies, bacterial cellulose leads to creating a microenvironment that leads to neuron and peripheral nerve regeneration. It was demonstrated for the first time in neural tissue engineering that 3D bacterial nanocellulose (BNC) scaffolds cultured neuroblastoma cells (SH-SY5Y), proliferate, adhere, and also differentiate into mature neurons, as determined by functional action potentials detected by electrophysiological assays [87]. The proliferation, adhesion, and formation of 3D neuronal cell networks on 3D bacterial nanocellulose scaffolds can be further enhanced by cationic modification of this material, i.e., on trimethyl ammonium beta-hydroxy propyl cellulose, as demonstrated on PC12 cells, a widely-used model of neurons [88]. In addition to their potential application in neural tissue renewal, constructs made of nanocellulose-based neural tissue were created and used as innovative tools for brain research. In order to achieve this, electrically conductive scaffolds are 3D printed using cellulose nanofibrils and carbon nanotubes generated from wood, which encourage the proliferation, adhesion, and differentiation of human neuroblastoma cells (SH-SY5Y) [89]. In fact, using nanocellulose to treat neurological diseases can increase proliferation and maintain the activity of neural tissue-derived cells. With further development of science in this field, due to the biocompatibility of cellulose, these materials can be used as composites and scaffolds to transfer neural tissue-derived cells to patients. Due to the structural support of nanocellulose s for the proliferation of these cells, its use will be more efficient than cell transfer alone. It is suggested that such studies be performed first in animals with neurological impairment, including induced autoimmune encephalomyelitis in mice, to obtain more and better information on the therapeutic efficacy of this structure. However, the therapeutic use of nanocellulose in neurological diseases is in its infancy.

### 3.2 Cartilage tissue

**Fig. 1:**
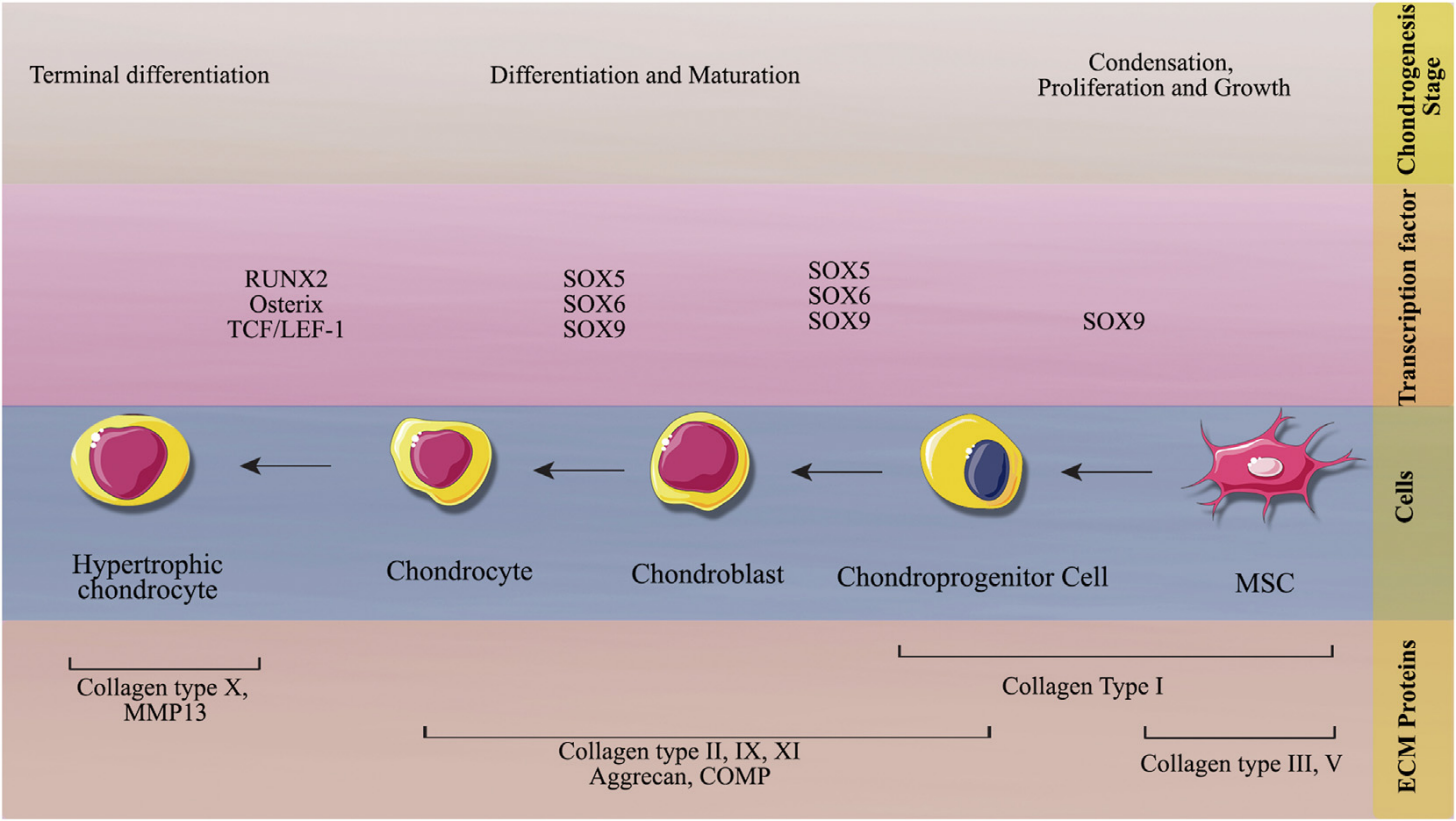
**Nanocellulose therapeutic application and its effects on mesenchymal stem cells for treating cartilage-related diseases.** The use of nanocellulose-based substrates and scaffolds enhances chondrogenic differentiation and expression of extracellular matrix-associated genes in MSCs and makes them better sources for use in cartilage-related diseases.

Pharmacological medications for pain relief and stiffness reduction are among the treatments for articular injury; surgery is chosen to treat more severe cartilage injuries [90]. Nanocellulose (NC) is the most promising candidate for biomedical tools in contact with human cells [53]. This is divided into three categories, namely cellulose nanocrystals (CNCs), nanofibrillated cellulose (NFC), and bacterial nanocellulose (BNC) [91]. Because it has intrinsic biocompatibility and biodegradability [91]. In the area of biomedicine, nanocellulose can be utilized to create synthetic scaffolds that replace ligaments and tendons, matrices for 3D cell culture, and grafting materials for dental implants [92]. Making stable and functional auricular cartilage tissue also depends on the cell source used [92] (Fig. 1).

Since cartilage is an avascular tissue, it cannot heal and/or regenerate [93]. The main barriers for scientists include guaranteeing that MSCs have the proper differentiation and matrix synthesis while preventing the formation of fibrocartilage or the development of hypertrophic cartilage, which may eventually ossify [94], and available reconstructive options for cartilage repair or replacement are limited [92]. Bioprinting technology is a recent novel cartilage replacement option [94]. It can generate 3D tissue via the layer-by-layer deposition of a scaffold structure containing its appropriate cell type [95]. Using the right bioink, which has the necessary viscoelastic properties for printing quality and long-term structural stability, is essential to creating printable structures [96]. As an alternative to bioink, there is the possibility of generating multiple-layer scaffolds [92]. Nanocellulose-based scaffolds have also been researched for ligament, muscle, and cartilage tissue engineering [97]. These tissues differ significantly from other organs [98]. Tuning the NC scaffolds in a stable condition is an important issue [99]. Maximum elastic modulus and strain of Human ligaments in the 100s of the MPa range and 20–30 %, respectively [99]. Age, gender, and physical activity affect this issue [99]. Similar mechanics were found in partially regenerated CNF composites via sequential dissolution in ionic liquids and hot pressing by a recent publication prepared [100]. The articular cartilage system is relatively avascular and there are outstanding challenges for revascularization of this tissue type [91,92]. This makes it a common target in developing materials for tissue engineering due to the prominent challenges associated with revascularizing a tissue [92]. Articular cartilage has a very limited capacity to repair itself [99]. Because it is composed mostly of an extracellular matrix, with a few chondrocyte cells distributed throughout its tissue [99]. Scaffolds that can withstand mechanical stresses of up to 10 MPa and have pore diameters between 200 and 300 μm are therefore beneficial in this field [99]. It has been established that the bilayer BNC scaffolds, which are non-cytotoxic and non-pyrogenic, have good mechanical stability and preserve structural integrity in addition to offering a porous architecture that promotes cell ingrowth [101]. The large surface area of NC and its high volume ratio, allowing for the adsorption of a variety of molecules, ions, and atoms and higher water uptake capacity, is the main potential of NC in cartilage tissue engineering [101,102]. Also, the cell adhesion mechanism enables cells to adhere to cellulose using the nanocellulose's hydrophilic hydroxyl moieties [102]. Moreover, the mechanical strength and high water-retention capacity of NC led to the further development of this type of cellulose application for auricular cartilage tissue regeneration in cartilage tissue engineering [103]. Due to its closeness in terms of host tissue response and mechanical strength to auricular cartilage, bacterial nanocellulose with a 17% increase in cellulose concentration was discovered to be a promising non-resorbable biomaterial for auricular cartilage tissue engineering [104]. Bilayered scaffolds made of alginate and bacterial NC were another promising material for this application. They were non-cytotoxic and non-pyrogenic and stimulated the proliferation of human nasoseptal chondrocytes [105]. Human femoral condyles covering cartilage-derived chondrocytes were cultured on bacterial NC scaffolds modified by laser perforation as substrates for articular cartilage engineering. These new scaffolds improve and promote nutrient transport, chondrocyte ingrowth and differentiation, and deposition of freshly produced extracellular matrix [106]. The use of a bioink based on nanocellulose for 3D bioprinting using living cells was another innovation. A wood-derived nano-fibrillated cellulose bioink containing human articular chondrocytes was used to 3D print anatomically developed cartilage structures, including a sheep meniscus and a human ear [107]. For 3D printing, a comparable bioink was combined with human chondrocytes that had been exposed to radiation and induced pluripotent stem cells (iPSCs), both of which were obtained from articular cartilage [108]. Bovine chondrocytes isolated from femoral condyle cartilage were stimulated in their spreading, proliferation, and synthesis of collagen II by an alginate sulfate/bacterial nanocellulose bioink [109]. A double cross-linked interpenetrating polymer network comprising sodium alginate and gelatin hydrogels, supplemented with 50 % wt of cellulose nanocrystals, was another intriguing composite material created for cartilage tissue engineering [100]. The use of nanocellulose in the treatment of intervertebral disc degeneration is also promising. Gellan gum hydrogels supplemented with cellulose nanocrystals were developed as substrates for regenerating the annulus fibrosus, or the disc's outer layer [110]. Considering the nature of the cartilage system using the scaffolds would be a great help for this field. Further studies are recommended to expand the available knowledge about the scaffolds and its potential in cartilage damage treatments. Meanwhile, the nanocellulose and nano-lignin had been less studied. Considering the role of the nanocellulose and nano-lignin as the next generation of the scaffolds more studies are recommended to get more reliable results in this field.

### 3.3 Vascular disease

In various scenarios, the arteries can get clogged or severely injured [111]. Using veins derived from the patient's body/donor has some disadvantages, as previously discussed in the bone tissue section. Tissue engineering may be able to assist us. BNC can be utilized to generate tubular structures in vascular tissue engineering. The benefits of BC application as artificial vessels include the absence of cell alterations, thrombus-free patency, acute inflammatory reaction symptoms, and the confirmation that it is a perfect material for manufacturing because of its superior tear resistance tubes, form retention ability, and moldability [112]. By adjusting the chamber diameter and bacterial cultivating period, we may control the thickness and size of BC by constructing BC tubes based on a planar BC membrane, which is very adaptable [113]. Another way to mimic tubular structures in the body is to create multilayered tubes out of the planar BC membrane. The excellent execution of that is demonstrated by the active growth of cells in the BC tubular constructs in vitro for an extended period without thrombosis. Many bypass procedures are conducted each year throughout the world. Arteries have been taken from patients' legs or thorax [114]. According to certain studies, In experimental animal models, BC has been employed effectively as a blood vessel for microsurgery. Silicone tubes are used as molds to construct tubular structures made of bacterial nanocellulose in vascular tissue engineering. These tubes were also considered to have excellent potential for substituting other hollow organs, including the ureter and the esophagus [115,116]. In a study by Weber et al., bacterial nanocellulose tubes were used in sheep to replace the right carotid artery. After explantation, the histologic analysis doesn't reveal any acute signs of foreign body reaction, such as acute inflammatory responses or immigration of undesired cells. It, therefore, provided evidence for the excellent biocompatibility of these tubular structures. Nevertheless, individuals undergoing implantation of these synthetic tubes have to get antiplatelet medication due to their significant risk of thrombotic blockage [115]. A further intriguing notion was coating endovascular stents using bacterial nanocellulose coupled with superparamagnetic iron oxide nanoparticles. This method increases the attraction of vascular smooth muscle cells (VSMCs) for in situ reconstructions of the blood vessels' tunica media. In the in vitro experiments, polyethylene glycol-coated magnetic bacterial nanocellulose forms suitable scaffolds for porcine VSMCs, showing supportive effects on migration, cell viability, and minimum cytotoxicity. This material also represents suitable mechanical properties and is considered promising for treating brain vascular aneurysms [117,118]. NC scaffolds were also used in vasculogenesis studies. Bacterial NC scaffolds combined with IKVAV (Ile-Lys-Val-ala-Val) peptide (integrin ligand derived from laminin) that used for human melanoma SK-MEL-28 cells vasculogenic mimicry studies. They seemed to offer a potential three-dimensional platform for anticancer drug screening [119]. Bacterial NC additionally demonstrated tremendous promise for bone TE even in its unmodified state. The adhesion, multilayered growth, and osteogenic differentiation of bone marrow MSCs obtained from rat femur were induced by bacterial nanocellulose in the absence of additives. Second Harmonic Generation (SHG) imaging demonstrates this; MSCs on bacterial NC scaffolds generated a mature form of collagen I and demonstrated alkaline phosphatase activity [120]. In a study on pigs, 10 mm long BNC grafts with a diameter of 3.0–3.7 mm were used, and the wall thickness of each of these BNC tubes was reported to be 0.6–1.0 mm [121]. This study shows that replacing the carotid artery in pigs with these cataracts for three months accelerates the formation of stable vascular canals in these animals. However, consistent mechanical adaptation between the implanted arteries and surrounding cardiovascular tissue is essential to prevent intimate hyperplasia (increased cell proliferation leading to severe enlargement of an organ) and failure of transplant replacement [122]. Therefore, the PVA-BNC composite, which is entirely compatible with the aorta in the physiological range and has a wide range of mechanical properties, is used for grafting [123]. Thus, some of the chemical changes made to nanocelluloses significantly increase their therapeutic efficacy. However, bacterial NC is more widely used in these diseases. It is suggested that in future studies, several methods be used to increase the therapeutic efficacy of NC in vascular transplantation and compare the results with each other to select the best approach. Laboratory animals with similar vascular damage can be used for this purpose. The results of these studies can be translated into clinical studies and can be of great help in this regard.

### 3.4 Bone repair

Bone-related TE consists of combining cytokines, scaffolds, and cell seeding [124]. The goal of this procedure is to replace the damaged area with bone TE scaffolds in order to treat the bone defect [124]. Successful intracellular movement, nutrient delivery, and extracellular matrix formation require a well-designed structure with high porosity, high interconnectivity, and defined pore size and pore geometry [16,98,[125], [126], [127]]. A higher life expectancy has the high incidence of diseases that cause bone loss, such as tumors, infections, and trauma [128]. Because of this, optimizing bone graft technology is crucial [129]. This necessity is highlighted in older people, where bone turnover is significantly decreased compared to young people [130]. A beneficial grafting material should contain (1) Development and differentiation of osteoblasts, the primary cellular constituent of bone [130]; (2) Bone growth direction in the appropriate areas [131]; (3) Integrating the new growing bone into the surrounding bone [132]; (4) Calcium phosphate crystals and essential elements of bone deposition [133]. In practice, it represents the ideal grafting materials [134]. Nevertheless, there are certain disadvantages to synthetic Ha related to its chemical and mechanical stability [135].

**Fig. 2:**
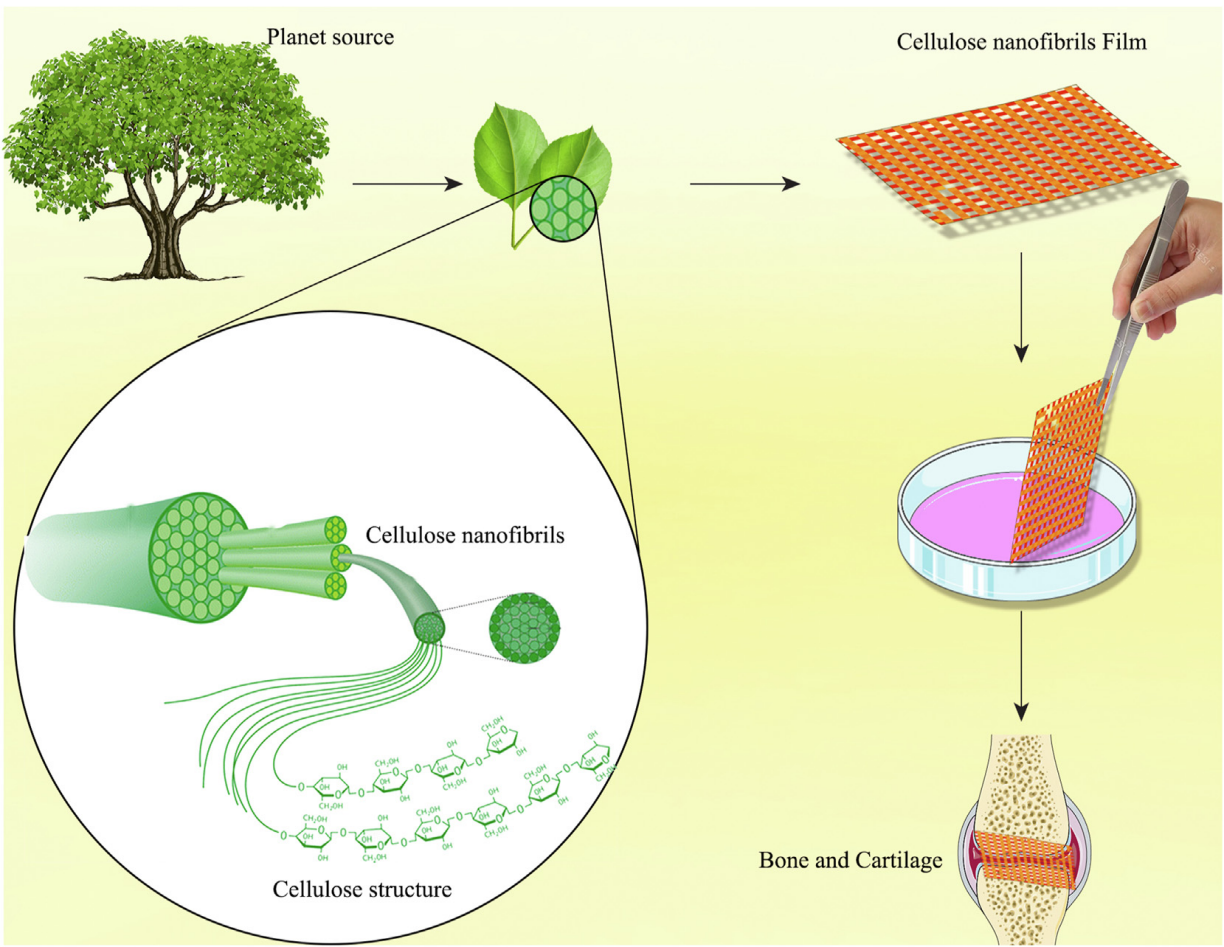
**Plant-derived nanocelluloses for the treatment of bone diseases**.

Additionally, nanocellulose is becoming a more and more attractive solution for developing novel and effective bone grafting techniques [130]. Applying and using bacterial nano cellulose is a new bone repair procedure, as traditional methods such as bone grafting have limitations such as immunological rejection and disease transfer, among others [93]. Bone is a mineralized connective tissue that is made up of organic and mineral materials such as collagen and nano-collagenous proteins [73]. Collagen and a solid matrix of inorganic calcium phosphates surround osteoblasts, osteocytes, and lining cells in bone tissue. Wood-derived nano-fibrillated cellulose is also used to construct scaffolds for bone tissue engineering, and their efficacy is proved on human MSCs grown on cellulose and chitin-containing composite scaffolds [127] (Fig. 2). The performance of mesenchymal stem cells and other bone-forming cells, such as rat calvarial osteoblasts, on nanocellulose-based scaffolds can be improved by biomimetic mineralization with calcium phosphates as hydroxyapatite and tricalcium phosphate [136,137]. According to this research, BNC, an operational scaffold produced by *Glucoacetobacter xylinus*, makes bone regeneration easier. In addition, to increase their efficacy, these scaffolds can be coupled with collagen I or osteogenic growth peptides [138]. Nanocellulose is also promising for bone implant coating. A hybrid coating, consisting of 45S5 bioactive glass individually wrapped and interconnected with fibrous cellulose nanocrystals (CNCs), was deposited on 316L stainless steel to strengthen bone-to-implant contact and accelerate the bone healing process. This coating substantially accelerated the proliferation, spreading, attachment, and differentiation of mouse osteoblast progenitor cells (MC3T3-E1) in vitro and mineralized the ECM deposited by these cells [139].

Similarly, scaffolds that coat 3D-printed polycaprolactone with wood-derived hydrophilic cellulose nanofibrils enhanced the proliferation, attachment, and osteogenic differentiation of human bone marrow-derived MSCs [140]. Several studies evaluated using these scaffolds and their efficacy. In a rabbit model, Urethral reconstruction was performed by using 3D porous bacterial cellulose scaffolds seeded with rabbit lingual keratinocytes. Also, a dog model using smart bilayer scaffolds comprising a nanoporous network of bacterial cellulose and a microporous network of silk fibroin [135,141]. These bilayer scaffolds were pre-seeded with dog lingual tissue obtained by biopsy-isolated keratinocytes and smooth muscle cells. The microporous scaffolds supported the growth and penetration of smooth muscle cells, while the nanoporous network provided good support for epithelial cells. Bone and cartilage have different defects associated with diseases, injuries, and aging [142]. Some of these defects can be treated by using autografts, allografts, and xenografts; however, their general use is affected by immune rejection [143]. According to the broad spectrum of bone and cartilage disorders, scaffolds can be a great help in this type of human disease. Despite the significant role of nanocellulose and nano-lignin as new scaffolds, they have been less studied. Available publications are focused on the possible role of these types of scaffolds as the repairing option for human bone and cartilage systems. More multicenter studies are recommended to investigate the ability of these brand-new scaffolds.

### 3.5 Skin disease

Some cellular and biochemical components are essential in the repair of injured tissue. Homeostasis/inflammation, proliferation, and remodeling are the three stages of wound healing [144]. The emergence of new substrates focusing on nanotechnology has increased interest in their use in healing skin wounds. Wound healing is a natural biological process that maintains the integrity of the skin and reduces the damage caused by injury [145]. The skin creates an effective barrier against the external environment, so the rapid healing of wound tissue after the injury is of particular importance. Four basic mechanisms, including activation of coagulation, inflammation, proliferation, and tissue regeneration, are commonly involved in wound healing [146]. In all four stages of wound healing, BNC aids in speeding up the healing process. In 1990, bacterial cellulose was first used for wound healing [147]. BNC is now used to treat infected wounds, traumatic injuries, diabetic wounds, second and third-degree burns, ulcers, abrasions, pressure sores, venous stasis, ischemia, lacerations, and biopsy sites, as well as various skin ailments. Wound dressings must have particular features to operate well; BNC has exceptional properties that make it a suitable candidate for wound dressing production [148]. Some of the most important properties include keeping the wound bed moist, high exudate absorption ability, adhesion to the wound bed, less irritation, antibacterial activity, ease of handling and exchanging (removal can cause trauma and damage to the wound site in traditional wound dressings), permeability for substances, reduced infection, avoid allergic reactions, and reduce wound pain, among others. All of these characteristics are advantageous to skin restoration. In a nutshell, the BC creates the optimal environment for the wound to heal as rapidly as possible while avoiding infection. Traditional wound dressings have several disadvantages, including the inability to retain moisture and the inability to stimulate wound healing. BC has been shown to regenerate more epidermis and dermis thickness, regulate angiogenesis and connective tissue creation and express more collagen, all of which help to expedite wound healing. The physical alteration, microbial fermentation, chemical modification, and compound modification can all be used to improve the qualities of the wound dressing. In general, the current facilities can be concluded [148]. Many different factors, including different cell populations, soluble mediators including cytokines and various growth factors, and extracellular matrix (ECM) components, are involved in this process [149]. During severe wounds and severe injuries to the skin, including diabetic wounds and second and third-degree burns, the wound-healing process occurs slowly and with a very low probability of leading to complete tissue repair [150]. Therefore, it is very important to use treatments to help wound healing and speed up its process. Many research groups use these particles to achieve this important goal due to nanoparticles' desirable and unique properties [151]. Polymers composed of natural resources became more popular than synthetic materials due to safety, economic, and environmental concerns in pharmaceutical applications, which led to innovative advances in biodegradable sources. Natural resource-derived polymers can mimic ECM properties to help cell proliferation and differentiation at the use site [152,153]. Lignin is one of the most abundant and important natural substances in biomass, which is used as a biopolymer in various treatments [154]. Also, in order to wounds repair in injuries such as burns, the use of biological substances that have properties such as non-toxicity, keeping the skin moist and antimicrobial is very important and can accelerate the healing process. Nanocellulose-based hydrogels are a new compound that has received significant attention for wound dressing [155,156]. These hydrogels have the mentioned properties and can be hydrated by imitating biological tissue and porous structure [157]. The results of a study conducted by El-Hoseny et al. Shows that using hydrogels containing nanocellulose in only a period of 3 weeks with excellent healing with 70 % wound closure [158]. In addition, the use of bacterial nanocellulose-based composites in very severe burns has been shown to reduce pain in patients and complete wound healing within two months after burns [159]. Drug delivery plays an important role in continuously delivering drugs or essential components, including antimicrobials, to the wound site [160]. A drug delivery system is a biotechnological engineering system that delivers therapeutic agents in a better way, to the target organs and tissues. In another study, thymol was added to these materials to increase the performance of BNC-based composites and hydrogels. The results of this study show that the use of this hydrogel increases the proliferation of fibroblasts at the wound site, increases their survival, and reduces cytotoxicity. As a result of comparing the performance of BNC hydrogel with thymol BNC combined hydrogel, the combined hydrogel with the ability to heal wounds in 25 days has a better performance than BNC hydrogel [161]. Therefore, it is recommended to use other drugs in hydrogels or composites to treat diseases by nano cellulose to increase the treatment efficiency. Another method that can be used for wound healing in nanocellulose-based tissue engineering is to add nanocellulose composites and hydrogels. Due to the importance of skin fibroblasts in repairing skin tissue and its function, the use of these cells can be precious [162]. In a study by Lohe et al., They used a biodegradable composite composed of BNC and acrylic acid to transfer human skin fibroblast (HDF) cells to evaluate their performance in healing wounds in atomic mice. Examination of the wounds at three times on day 1, day 7, and day 14 shows that using 50,000 HDF cells leads to complete wound closure after 14 days [161]. The healing rate is higher in the early stages, leading to 70 % wound healing. Further analysis shows that more than 50 % of the HDF cells in the composite move to the wound site within 24 h. In addition, the analysis and comparison of the microstructure of the newly formed skin after treatment with engineered hydrogel with natural skin resulting from non-intervention repair did not show a significant difference. Another method is to use NC in the capsule of drugs to maintain stability to ionic changes, pH, temperature around the target help [161]. Well known, wood-derived NC, by incorporation with various compounds and irons, have antimicrobial effect. Plant nanocellulose has been used for wound dressing applications partly based on its capability to form translucent films with good liquid absorption capabilities. Some plant derived NC materials such as 2,2,6,6- tetramethylpiperidinyl-1-oxyl (TEMPO)-mediated oxidation is usually applied as a pretreatment to produce Cellulose nanofibrils. Cellulose nanofibers could be arranged as thin films, which are transparent, water absorbent and vigorous. These properties make it a best candidate to be used as scaffolds for tissue regeneration/engineering [[163], [164], [165]] or as a wound dresser [166,167]. Consequently, it's critical to evaluate how liquid absorption affects the films' resistance and provide additional insight into their behavior in wound care scenarios. Thus, it is important to assess the tensile properties and elasticity of cellulose nanofibril dressing film in wet conditions. Since success in wound management depends on the ability of NC dressing to keep a moist environment in the wound.

## 4 Conclusion

Today, there is a great interest in using biomass as a source of energy and renewable materials. NC, cellulose in the form of nanostructures, has been proven to be one of the most prominent green materials of modern times. This term covers a wide range of cellulose-derived materials with at least one dimension in the nanometer range with many desirable properties. For this reason, these substances are used to treat many diseases. Although NC has various salient and desirable properties and is widely available in nature, extracting NC from lignocellulosic biomass or cellulosic materials is still a significant challenge. Due to the accumulation of lignin, hemicellulose, and other substances in the plant cell wall, biomass pretreatment is an essential step to remove all non-cellulosic materials for therapeutic applications of these bionanomaterials. However, such a process always has very complex steps. It is associated with the production of acidic wastewater for acid hydrolysis, prolonged reaction time for enzymatic hydrolysis, and high energy consumption for the mechanical process. Thus, the design and progress of novel methods that can extract cellulose with high purity and increased safety are important for clinical use. Besides, NC's superior mechanical strength and biocompatibility benefit cardiac TE. The excellent shear thinning, shape fidelity, and extrudability of NC-based bioinks are conducive to liver TE, which requires complicated 3D structures.

However, NC is nearly indegradable in vivo despite its adaptability, which has an impact on the organism's absorption. Consideration should be given to improving degradability by techniques including cutting down on NC's crystallinity and controlling the pace of degradation, as these are essential factors in the applications of NC-based scaffolds, particularly in vascular and skin TE. Meanwhile, because of its broader uses in multiple dimensions, functionalizing NC by grafting a polymer onto its backbone is also an intriguing approach. Potential nanoparticles that can be used as reinforcing agents in different nanocomposites are made possible by these alterations. They also promote specific features for producing novel cellulosic nanomaterials, aiming to promote their applications in the field of functionalized nanomaterials.

## Funding

This study was financially supported by a grant from the Stem Cell Research Center at Tabriz University of Medical Sciences (Grant No. 56951).

## Availability of data and materials

The data supporting the conclusions of this article are all online.

## Ethics approval and consent to participate

This study was conducted with the ethics code (IR.TBZMED.VCR.REC.1399.224) at Tabriz University of Medical Sciences. approval.

## Authors contribution

**KM** and **AH**: Conceptualization, Investigation, Writing - original draft, Writing - review & editing, Validation, Supervision, Edited final version of the manuscript. **AKH**: Writing - original draft, Supervision, and final approval of the manuscript. **MA**: Supervision and final approval of the manuscript. All authors contributed to the article and approved the submitted version.

## Consent for publication

Not applicable.

## Declaration of competing interest

Please declare any financial or personal interests that might be potentially viewed to influence the work presented. Interests could include consultancies, honoraria, patent ownership or other. If there are none state ‘there are none’.
